# Novel artificial intelligence for diabetic retinopathy and diabetic macular edema: what is new in 2024?

**DOI:** 10.1097/ICU.0000000000001084

**Published:** 2024-09-09

**Authors:** Stela Vujosevic, Celeste Limoli, Paolo Nucci

**Affiliations:** a Department of Biomedical, Surgical and Dental Sciences, University of Milan; b Eye Clinic, IRCCS MultiMedica; c Department of Ophthalmology, University of Milan, Milan, Italy

**Keywords:** artificial intelligence, deep learning, diabetic retinopathy, foundation model, retinal imaging

## Abstract

**Purpose of review:**

Given the increasing global burden of diabetic retinopathy and the rapid advancements in artificial intelligence, this review aims to summarize the current state of artificial intelligence technology in diabetic retinopathy detection and management, assessing its potential to improve care and visual outcomes in real-world settings.

**Recent findings:**

Most recent studies focused on the integration of artificial intelligence in the field of diabetic retinopathy screening, focusing on real-world efficacy and clinical implementation of such artificial intelligence models. Additionally, artificial intelligence holds the potential to predict diabetic retinopathy progression, enhance personalized treatment strategies, and identify systemic disease biomarkers from ocular images through ‘oculomics’, moving towards a more precise, efficient, and accessible care. The emergence of foundation model architectures and generative artificial intelligence, which more clearly reflect the clinical care process, may enable rapid advances in diabetic retinopathy care, research and medical education.

**Summary:**

This review explores the emerging technology of artificial intelligence to assess the potential to improve patient outcomes and optimize personalized management in healthcare delivery and medical research. While artificial intelligence is expected to play an increasingly important role in diabetic retinopathy care, ongoing research and clinical trials are essential to address implementation issues and focus on long-term patient outcomes for successful real-world adoption of artificial intelligence in diabetic retinopathy.

## INTRODUCTION

Diabetic retinopathy is a rapidly growing global health concern, and early detection is crucial for effective management [1]. In the past decade, diabetic retinopathy has emerged as a frontrunner field for artificial intelligence ophthalmic applications, enabling rapid advances in clinical care, scientific research, and medical education.

The aim of this review is to summarize the current state of the emerging technology of artificial intelligence in diabetic retinopathy to assess its potential to improve care and visual outcomes in real-world settings and discuss multifaceted aspects of artificial intelligence implementation across the globe. 

**Box 1:**
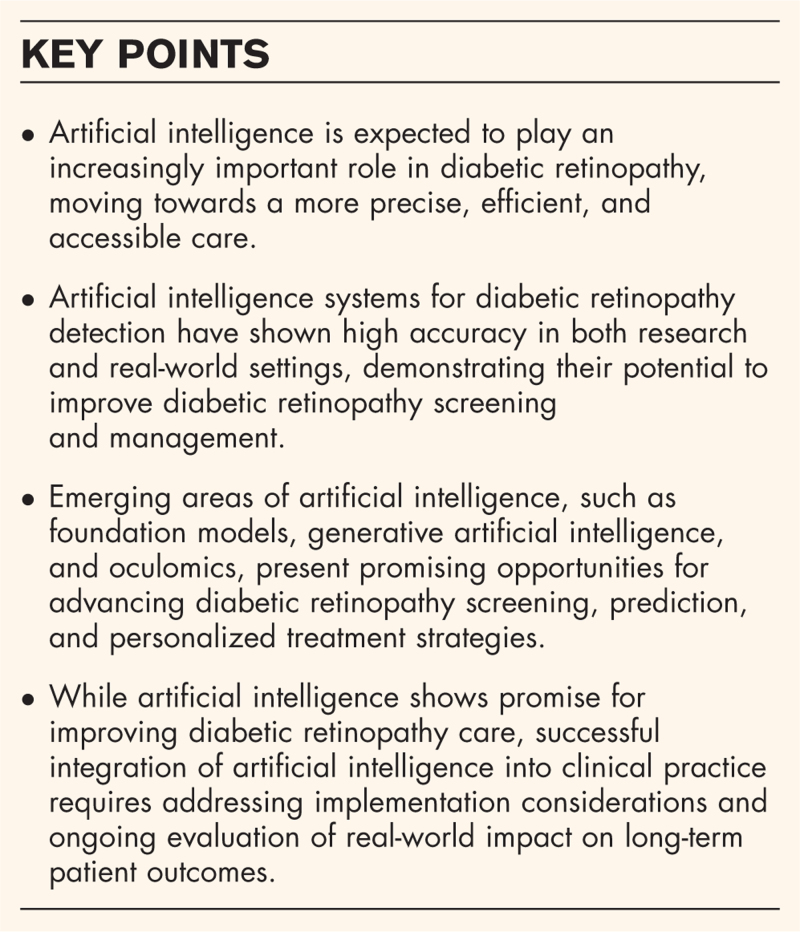
no caption available

## UNIMODAL ARTIFICIAL INTELLIGENCE FOR RETINAL IMAGE ANALYSIS

### Automated DR screening

Since the approval of the first-ever artificial intelligence based device for the automated early detection of diabetic retinopathy in 2018 by the Food and Drug Administration (FDA) [2], the use of artificial intelligence in diabetic retinopathy has grown exponentially positioning it as the second most utilized procedure for artificial intelligence in the United States (US), second only to coronary artery disease [3^▪▪^].

Medical artificial intelligence models may equal or even outperform accuracy of clinicians in image-based tasks for automated diabetic retinopathy screening, grading, and point-of-care (POC) testing [4^▪^]. Thus, developing a streamlined artificial intelligence assisted diabetic retinopathy screening workflow could improve accessibility and detection accuracy while addressing healthcare provider shortage, potentially leading to better visual outcomes.

However, high in-silico performance of artificial intelligence does not ensure its performance *in vivo* and a real-world impact. Thus, there has been increasing emphasis on evaluating clinical applicability and expected efficacy of artificial intelligence algorithms for diabetic retinopathy screening in real-world settings to bridge the gap between artificial intelligence technology in research settings and actual clinical practice [5–11] (Table 1).

**Table 1:** Performance of artificial intelligence systems for the detection of diabetic retinopathy in real-word settings (selected studies)

Ref.	Type of study	Population/setting	No. of diabetic patients	Output	Sn/sp %	Camera	AI system	Image acquisition for automatic DR detection
Table-top fundus camera								
Skevas *et al.*[5]	Prospective study	Real clinical environment in Germany	231	rDR (more than mild NPDR)	100/80.1	Table-top fundus camera (DRS Plus, Icare Finland Oy, Finland)	DLS for DR, AMD and glaucoma	Mydriatic macula-centered 45° image Study nurse
Whitestone *et al.*[6]	First clinical study on AI-based screening for DR in Africa	Four diabetes clinics in Rwanda	827	rDR	92/85	Table-top fundus camera (Topcon NW400, Tokyo, Japan)	DLS based on the Inception-ResNet-v2 CNN architecture	Nonmydriatic 45° macula-centered and optic-disc-centered images Trained personnel
Chia *et al.*[7]	Retrospective external validation study	Indigenous primary care service in Australia	864	mtmDR vtDR All cause rDR	98.0 / 95.1 96.2 / 95.8 93.7 / 91.7	Table-top fundus camera (Maestro, Topcon Co. Tokyo, Japan)	DLS - deep neural network trained with an ‘Inception-V.4’ architecture	Nonmydriatic, single-field, 45°, macula-centered images
Handheld fundus camera								
Salongcay *et al.*[8]	Prospective comparative study	Community-based DR screening program in Philippines	2793	rDR vtDR	86 / 86 92 / 80	Handheld fundus camera (Aurora IQ, Optomed Ltd)	Integrated AI algorithm SELENA Eyris DLS	Mydriatic macula-centered and optic-disc-centered 50° images Trained and certified retinal imagers
Lupidi *et al.*[9]	Prospective, observational cross-sectional study	First-line DR screening in a real-world clinical setting in Italy	256	DR (any sign)	96.8 / 96.8	Handheld fundus camera (Optomed Aurora, Optomed, Oulu, Finland)	Integrated AI algorithm SELENA Eyris DLS	Non mydriatic, macula-centered 50° image Single operator
Smartphone-based fundus camera								
Kemp *et al.*[10]	Clinical validation study	Real-world DR screening program in a Caribbean population in Dominica	587	rDR (≥ moderate NPDR or DME)	77.5 / 91.5	Smartphone-based fundus camera (Model FOPNM-10, Remidio Innovative Solutions, Bangalore, India)	Medios DR AI software (NM App V.2.0, Mediostech, Singapore)	Mydriatic macula-centered and optic-disc-centered images Field grader
Malerbi *et al.*[11]	Multicenter cross-sectional diagnostic study	Three diabetes care and eye care facilities in Brazil	327	Any DR mtmDR	90.5 / 90.6 90.2 / 85.1	Smartphone-based fundus camera (The Eyer, Phelcom Technologies, LLC)	Two DLS -Modified versions of the CNN Xception and EfficientNetV2S CNN	Mydriatic 45° macula-centered images Trained healthcare professionals

AI, artificial intelligence; AMD, age-related macular degeneration; CNN, convolutional neural network; DLS, deep learning system; DME, diabetic macular edema; DR, diabetic retinopathy; NPDR, non proliferative DR; rDR, referable DR; Sn, Sensitivity; Sp, Specificity.

A recent meta-analysis of 34 population-based studies evaluated the performance of different retinal photography modalities, including tabletop, handheld, and smartphone-based fundus cameras for diabetic retinopathy screening in healthcare practice [4^▪^]. The analysis showed a high detection rate with a pool sensitivity of 94% and specificity of 89% when compared to human graders [4^▪^]. Notably, artificial intelligence accuracy remained substantial when applied to both mydriatic and nonmydriatic images, with the latter offering advantages, particularly in under-resourced settings [4^▪^].

Recent advancements in fundus camera technologies have led to a surge in the adoption of handheld and smartphone-based fundus imaging across various settings due to their accessibility, cost-effectiveness, and user-friendliness, as opposed to high-cost table-top retinal cameras [12,13].

The performance of handheld [8,9] and smartphone-based imaging devices [10–12] for automated diabetic retinopathy detection in real-life settings has yielded varying but generally high levels of diagnostic accuracy (Table 1). Of note, in April 2024, FDA approved the first fully autonomous handheld artificial intelligence diabetic retinopathy screening device [14]. Combining these portable devices with fully autonomous artificial intelligence might improve POC diabetic retinopathy screening, especially in primary care, low and middle-income countries (LMICs), and remote areas with limited resources [12,13].

Ultrawide-field (UWF) retinal imaging is another modern imaging modality that is increasingly being adopted in clinical settings and large-scale retinal disease screening due to its greater field of view of the retina (up to 80% of the retina area in a single, nonmydriatic photo) compared to traditional fundus photography [13]. Recently, DLS has demonstrated promising results in detecting retinal lesions on UWF images of Chinese patients in rural areas [15]. However, the cost of UWF imaging currently limits its clinical deployment in LMICs, and the performance of deep learning system (DLS) can be altered by factors such as low image quality, lesion size, and the complexity of lesion sets [15].

#### Cost-effectiveness

The accuracy of artificial intelligence (AI) models in diabetic retinopathy screening may not always translate to cost-effectiveness in real-world scenarios, so independent assessments are essential [13]. Recent analyses in China [16,17], Thailand [18], and Australia [19], have demonstrated the potential cost-effectiveness of artificial intelligence based diabetic retinopathy screening in real-world settings, supporting the adoption of deep learning (DL)-based diabetic retinopathy screening in primary care to promote a more efficient and equitable eye-care ecosystem.

Notably, the B-PRODUCTIVE trial in the US showed that an FDA-authorized artificial intelligence for diabetic retinopathy improves clinic productivity by 40% among patients with diabetes mellitus [20^▪^].

#### Towards clinical deployment of artificial intelligence in diabetic retinopathy

Although the demonstration of high diagnostic accuracy and cost-effectiveness of artificial intelligence based diabetic retinopathy screening aids in the justification for implementation, widespread clinical adoption of such artificial intelligence systems is still hampered by key challenges.

One major issue is the limited generalizability of artificial intelligence models beyond specific patient populations and settings used to train algorithms, potentially exacerbating healthcare disparities [21]. To ensure broad applicability and develop effective artificial intelligence driven clinical decision support systems, publicly available datasets for external validation are essential [21].

Additionally, poor image quality and false positives during diabetic retinopathy screening may lead to unnecessary tertiary referrals and increased healthcare costs. Artificial intelligence-human hybrid workflows, models prioritizing sensitivity and comprehensive representation of a range of disease severity are important for improved accuracy screening [17,22]. Quality assessment of fundus photos is also important for artificial intelligence model performance, fairness, and generalizability [23].

Furthermore, as artificial intelligence becomes better integrated into clinical practice, it is essential to evaluate the real impact of artificial intelligence implementation on screening rates, improving adherence and follow-up compared to traditional referral, and end-user acceptance. The ACCESS trial [24^▪^] found that using artificial intelligence led to significantly higher completion rates for eye exams among young patients with diabetes mellitus within 6 months(100%) compared to standard care (22%). This has been the first study to show the role of autonomous artificial intelligence in closing a guideline-based care gap. Notably, patients diagnosed with diabetic retinopathy by POC artificial intelligence were also more likely to complete the follow-up exams compared to those in the telemedicine program (Δ42%), possibly due to the shorter time to receive screening results.

Similarly, in another diabetic retinopathy screening program in the US, patients were three times more likely to attend ophthalmology appointments after receiving a positive diabetic retinopathy screening result from an artificial intelligence based workflow (69.2%) compared to both a human workflow (12%) and an artificial intelligence–human hybrid workflow (11.7%) [25].

Finally, a successful integration of artificial intelligence into clinical practice for diabetic retinopathy also requires ensuring equity and accessibility by targeting underserved, low-income, and racial/ethnic minorities to reduce disparities in eye care access and outcomes [26]. In this context, a clinical trial ongoing in Tanzania aims to implement an artificial intelligence system into an active diabetic retinopathy screening program to assess its diagnostic accuracy and impact on follow-up rates of people referred after screening [26].

Other implementation issues of artificial intelligence include data privacy, interoperability, training, algorithm bias, scalability, sustainability as well as ethical considerations and administrative barriers [27].

### Diabetic retinopathy prediction and risk stratification

Despite annual screening recommendations [1], personalized management of diabetic retinopathy is challenging due to the high variability in individuals’ disease onset and progression.

Dai *et al.*[28^▪▪^] developed and validated a DLS to prospectively predict individualized risk and time to diabetic retinopathy progression within 5 years using clinical metadata and 118 868 retinal images from 29 868 diabetic patients of different multiethnicity. Having achieved high accuracy in this task, this DLS underwent successful real-world validation in individuals with diabetes in China and India, extending the mean screening interval from 1 year to nearly 3 years [28^▪▪^]. The authors postulated that such an individualized risk model could be integrated into the clinical workflow to provide personalized diabetic retinopathy screening intervals aiming to improve screening efficiency and accessibility, particularly in resource-limited settings. This could lead to less delayed detection of diabetic retinopathy progression, and more targeted management strategies for those at high risk of progression.

Accurate classification of diabetic retinopathy severity is also essential for guiding effective treatment decisions and assessing disease progression. Wright *et al.*[29] developed interpretable DLS to categorize diabetic retinopathy severity stage accurately using just sex, age, and measures of visual function, including contrast sensitivity, microperimetry, dark adaptation, matrix perimetry, and color vision. This could be more sensitive in representing visual dysfunction in diabetes mellitus with the progression of diabetic eye disease and provide novel, noninvasive outcome measures based on real-world clinical data for assessing the efficacy of new treatments in clinical trials [29].

### Segmentation and feature detection of diabetic retinopathy

The discrepancy frequently observed between visual outcomes and anatomical improvements following therapy for diabetic macular edema (DME), coupled with the heterogeneous progression of diabetic retinopathy, advocates the need for personalized management strategies. While central retinal thickness remains the most relevant in DME management, additional structural parameters relevant to visual outcomes, disease activity, and prognosis, should also be considered to improve the personalized care of real-world patients with diabetes [30] Recently, an automated artificial intelligence algorithm has been proposed for the quantification of major OCT biomarkers in DME with the aim of providing an objective method for diagnosing and monitoring DME eyes within a personalized medicine approach [31].

A multiple instance learning-based network, MIL-ResNet, has been developed for classifying diabetic retinopathy features in weakly-labeled widefield OCT-angiography (OCT-A) images [32]. MIL-ResNet accurately detects biomarkers in OCT-A datasets, including neovascularization, microaneurysms, enlargement of the foveal avascular zone, and ischemic areas without requiring precise annotations. Hence, MIL-ResNet14 could serve as a reliable prescreening tool for early-stage diabetic retinopathy, assisting untrained personnel in clinical decision-making and enabling longitudinal disease monitoring.

### Beyond traditional medical tasks

An emerging field is represented by the application of artificial intelligence to extensive biomedical or image datasets to uncover novel disease biomarkers or predict different visual tasks, extending beyond conventional clinical applications.

A recent study [33] investigated the accuracy of an artificial intelligence algorithm in estimating Best-Corrected Visual Acuity (BCVA) from color fundus photographs (CFP) among eyes with DME. The model showed ‘not ideal’ performance in estimating BCVA with the best-performing algorithm's mean absolute error of Early Treatment Diabetic Retinopathy Study 9.7 letters. However, this artificial intelligence model has the potential to reduce the need for time-intensive manual refractions and visual acuity measurements, enabling cost-effective BCVA assessment in primary care and remote monitoring.

He *et al.*[34] developed and externally validated a diabetic retinopathy detection model based on the most important variables associated with diabetic retinopathy selected from 220 circulating metabolites and 19 risk factors. Machine learning algorithms identified diabetes duration, insulin usage, age, and tyrosine as the most important factors for diabetic retinopathy detection. Additionally, diabetic retinopathy was associated with hemoglobin A1c, blood glucose, pulse pressure, and alanine [34]. This approach could enhance the detection of diabetic retinopathy beyond the limitations of conventional risk factors alone.

## EMERGING AREAS OF ARTIFICIAL INTELLEGENCE IN DIABETIC RETINOPATHY

Foundation models and generative artificial intelligence are rapidly emerging in healthcare as a major revolution in artificial intelligence's capabilities, recognized as the third epoch of artificial intelligence evolution [35], yet applications in diabetic retinopathy remain relatively nascent.

### Foundation models

A significant breakthrough in ophthalmology has been the introduction of RETFound, a self-supervised learning-based foundation model for retinal images that outperforms traditional systems in image recognition tasks [36^▪▪^]. This model can provide a foundation for rapid adaptation in various tasks through transfer learning, with minimal labeled data for fine-tuning, reducing the need for extensive labeled datasets. RETFound [36^▪▪^] could correctly diagnose diabetic retinopathy and other sight-threatening ocular diseases by identifying disease-related patterns from CFP and also enhance the performance of oculomics tasks by predicting systemic diseases. Therefore, this innovative artificial intelligence model may promote incidence prediction and risk stratification of ocular and systemic diseases, highlighting its potential for effective community-based screening.

Recently, a RETFound-enhanced DL model [37] has been evaluated for multiple-eye disease screening using real-world images from community screenings. It outperformed commercially available models and offered a higher net benefit in community-based screening, supporting its adoption in LMICs.

### Generative artificial intelligence

Liu *et al.*[38] developed different *generative adversarial networks (GAN) models* to generate accurate posttherapeutic OCT images synthesized based on pretherapeutic OCT images of patients with DME. Using synthetic posttherapeutic OCT images to predict short-term treatment response could aid clinicians in designing optimal treatment strategies and better understanding the efficacy of antivascular endothelial growth factor treatment.

DL model based on *vision transformer* demonstrated relatively high accuracy for detecting diabetic maculopathy stages from OCT images, demonstrating potential for preliminary screening and therapeutic strategy development for patients with diabetes mellitus [39].

*Large language models (LLMs)* and *generative chatbots* for text generation, data extraction, and image data processing are potentially capable of answering patient and medical questions regarding ophthalmic knowledge and retinal disorders such as diabetic retinopathy [40,41]. Apart from analyzing text-based prompts, generative chatbots could also accurately respond to specific-domain questions based on OCT imaging interpretation [42]. Despite the rapid progress of LLMs and the promise of democratizing medical knowledge, careful regulation is necessary to ensure they effectively address real-life clinical and patient needs.

A study by Mohammadi *et al.*[43] demonstrated the use of ChatGPT and Vertex AI to preprocess CFP images, analyze data, and train machine learning models through a code-free method for diabetic retinopathy detection and severity assessment.

*Vision-language models* like ChatGPT-4 and Gemini Pro Vision can aid in detecting disease features on OCT images, making referral recommendations, and personalizing therapeutic strategies for macular disease by integrating knowledge from both text and image domains. Yet, due to their nascent stage of development, their capacity to interpret clinical ophthalmic images remains limited. Domain-specific fine-tuning could augment specialized vision capabilities of ophthalmic models to substantially improve artificial intelligence performance for highly specialized clinical tasks [42,44].

Other algorithms can perform image domain transformation between different imaging modalities.

Murata *et al.*[45] developed a convolutional neural network to infer fundus fluorescein angiography (FFA) images based on OCT-A, with the rationale of providing adjunctive information in OCT-A, specifically leakage patterns. The artificial intelligence inferred FFA accurately delineated vascular leakage and occlusion, achieving a structural similarity index of 0.91 with real FFA images.

Shi *et al.*[46] developed and validated a GAN model that could generate realistic venous and late-phase fundus FFA images from CFP. Adding translated FFA images to CFP improved diabetic retinopathy screening accuracy, suggesting it as an additional method for diabetic retinopathy screening or surrogate when FFA examination is not feasible.

### Code-free deep learning

CFDL or AutomL platforms are innovative tools that allow clinicians with limited or no coding experience to prototype novel clinical artificial intelligence applications, demonstrating promising potential in diabetic retinopathy care [47–49]. CFDL models have been developed and validated for identifying referrable diabetic retinopathy from handheld retinal images [49] and from UWF images for diabetic retinopathy progression prediction over time [50]. The potential role of CFDL may offer advantages over bespoke DL, reducing barriers and costs associated with artificial intelligence adoption for addressing clinical needs and advancing the democratization of artificial intelligence in healthcare. Its implementation should be carefully evaluated in comparison to bespoke DL models to prove its performance and clinical effectiveness in real-world scenario [47].

### Oculomics

Oculomics is a novel perspective in the field of noninvasive biomarkers for early detection of systemic diseases from ocular images [51].

Recent studies showed that DL algorithms can detect diabetic kidney disease from retinal photographs alone [52] across multiethnic populations, showing the potential as an adjunct to diabetic retinopathy screening.

Notably, features of ocular and systemic health are also contained in external eye images. A DLS system has been developed to detect signs of diabetic retinopathy, elevated glycated hemoglobin, and information about additional systemic parameters related to multiple organ systems and blood from photographs of the external eye [53^▪▪^].

By detecting these markers noninvasively at the POC and at low cost, oculomics holds the potential to be used as an accessible and noninvasive risk stratification tool to facilitate timely access to the correct disease management in a ‘precision medicine’ approach [51].

## CONCLUSION

Although many of these applications are currently in the research stages, artificial intelligence is expected to become increasingly integrated into diabetic retinopathy care in the coming years. Artificial intelligence implementation would offer a transformative approach to precision medicine and healthcare delivery. It will be essential for ophthalmology to embrace this rapidly advancing technology while establishing rigorous evaluation benchmarks and ensuring careful, patient-centered implementation to effectively utilize artificial intelligence for improving both patient care and the provider experience.

## Acknowledgements

*None.*

### Financial support and sponsorship

*This research was supported by the Italian Ministry of Health, Ricerca Corrente, IRCCS MultiMedica.*

### Conflicts of interest

*The authors report none conflict of interest related to this article.*

*This is an invited review by Drs Ting and Rahimy who are editing the Artificial Intelligence/Big Data section for the November 2024 issue of Current Opinion in Ophthalmology.*

## REFERENCES AND RECOMMENDED READING

Papers of particular interest, published within the annual period of review, have been highlighted as:

▪ of special interest
▪▪ of outstanding interest
